# Persistent Fever as the Solitary Manifestation of Familial Mediterranean Fever: A Case Report

**DOI:** 10.7759/cureus.108628

**Published:** 2026-05-11

**Authors:** Rayan Zaydan, Mais Alsadi, Khurshid Khan, Zaineb Benslimane

**Affiliations:** 1 Medicine, University of Sharjah, Sharjah, ARE; 2 General Pediatrics, Al Qassimi Women's and Children's Hospital, Sharjah, ARE; 3 Pediatrics, Al Qassimi Women's and Children's Hospital, Sharjah, ARE

**Keywords:** colchicine, familial mediterranean fever (fmf), fever of unknown origin (fuo), pediatric autoinflammatory disease, persistent fever

## Abstract

Familial Mediterranean fever (FMF) is an autoinflammatory genetic disorder characterized by prolonged periods of fever and serosal inflammation such as peritonitis, pleuritis, and arthritis. FMF is due to a mutation of the MEFV gene encoding pyrin. Although this disorder typically presents in early childhood with recurrent episodes that are self-limiting, atypical and long-lasting febrile episodes may cause significant diagnostic challenges, especially in regions where genetic testing for FMF is not easily available. In this study, we report a case of a four-year-old Syrian girl who presented with persistent fever unresponsive to antipyretics lasting for one month. With the high clinical suspicion, colchicine therapy was initiated, resulting in clinical improvement. This case illustrates the importance of maintaining clinical suspicion of FMF in the pediatric population presenting with persistent fever of unknown origin, and sheds light on the role of colchicine as a therapeutic and diagnostic medication when genetic testing is unavailable.

## Introduction

Familial Mediterranean fever (FMF), also known as periodic peritonitis, is a systemic autoinflammatory genetic disorder. It is named FMF due to the effect on Mediterranean and Middle Eastern individuals, including Armenians, Jews, Arabs, Kurds, Turks, Iranians, and Italians. FMF is characterized by recurrent episodes of fever associated with serosal inflammation of the abdomen, lungs, and joints, such as peritonitis, pleuritis, and arthritis [1]. It is a genetic disorder due to an autosomal recessive mutation of the MEFV gene located on chromosome 16, inducing innate immune activation in the body. FMF usually begins in early childhood before 20 years of age [2]. The disease occurs as recurrent episodes of fever that typically develop over a few hours and last anywhere from six hours to four days, then they tend to resolve spontaneously with no treatment. The bouts of fever could be accompanied by rash or unexplained headache. The diagnosis of FMF is challenging due to the disease being non-pathognomic and occurring in regions where the disease is uncommon. Therefore, if prolonged and undetected, FMF could induce more serious damage to the body, potentially causing kidney failure or other serious consequences [1]. The underlying cause is amyloidosis, which is the accumulation of abnormal protein deposits in various organs, such as the kidneys, leading to functional impairment. The primary treatment is colchicine, which plays a major role in preventing the development of these severe chronic manifestations [2]. This case stands out due to its unusual and prolonged presentation. Unlike the classical short, self-limited attacks of FMF, our patient presented with a constant one-month-long fever unrelieved by antipyretics. This case highlights the importance of considering FMF in pediatric patients with atypical presentations, especially in high-risk ethnic populations, even when the presentation deviates from the usual, classical pattern.

## Case presentation

**Figure 1:**
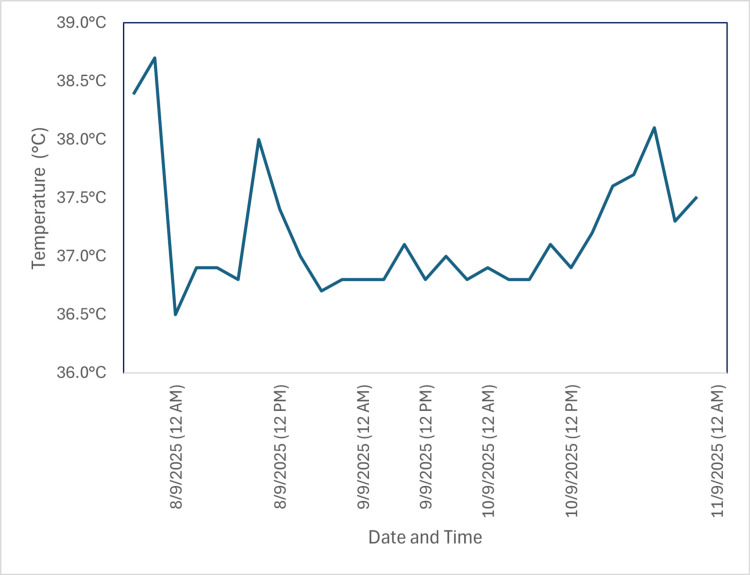
Temperature trends during the hospital stay. Highest and lowest temperatures (°C) recorded throughout each day during the three-day hospital stay.

A four-year-old Syrian girl was admitted on September 7, 2025, complaining of continuous fever ranging from 38°C to 39°C for a month. The fever would come on sporadically and persist for at least four to five hours, being resistant to antipyretics such as paracetamol or Voltaren. In addition, she reported left ankle pain for two days, intermittent abdominal pain a week ago, and chest pain that worsens with breathing, eating, or speaking. Moreover, she has been having night sweats and urinary frequency daily since then. She used to get this fever every one to two months, lasting for two days, for the past six months without any other respiratory tract symptoms. The parents were non-consanguineous, but the mother’s cousin had FMF managed with colchicine. On examination, she had pallor, mild splenomegaly, bilateral posterior cervical lymphadenopathy, pain over the center of the plantar side of the left foot, causing a limp, and generalized right-sided abdominal pain. Temperature was recorded continuously during the hospital stay (Figure 1).

**Figure 2:**
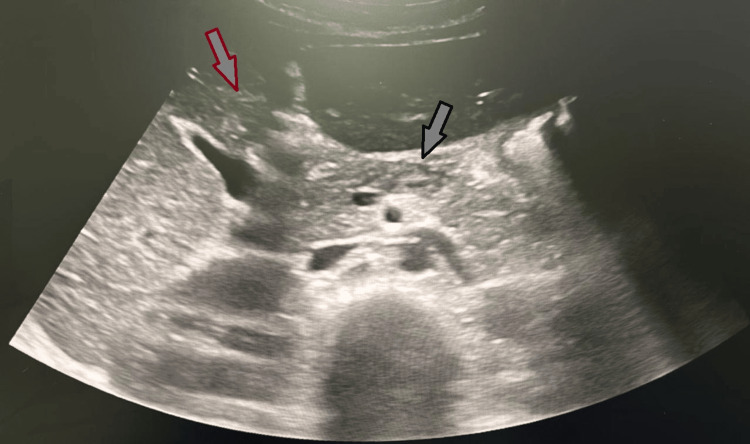
Transverse ultrasound of the abdomen. Ultrasound of the abdomen revealed mild splenomegaly (11 cm) and enlargement of the mesenteric lymph nodes in the right lower quadrant, while the liver and pancreas findings were unremarkable (black and red arrows, respectively).

On the day of admission, complete blood count revealed an elevated white blood count of 16.80 µL/mm³, low hemoglobin of 9.90 g/dL, and low mean corpuscular volume of 74.60 fL. The inflammatory markers were elevated with C-reactive protein at 110 mg/L and erythrocyte sedimentation rate at 54 mm/hr. The blood smear had neutrophilic leukocytosis with left shift, as well as a few reactivated lymphocytes. Urine analysis, stool analysis, blood culture, autoimmune profile, ankle X-ray, and soft tissue ultrasound were insignificant. In addition, an abdominal ultrasound was done, but the findings were not correlated with the presentation (Figure 2). Further investigations revealed elevated fibrinogen (5.85 g/L) and calprotectin (146.60 µg/g). The human leukocyte antigen B27 (HLA-B27) reaction came out as negative; however, her serum amyloid A (SAA) levels were markedly elevated (1580 mg/L) (Table 1).

**Table 1:** Lab investigations. MCV: mean corpuscular volume; MCH: mean corpuscular hemoglobin; ESR: erythrocyte sedimentation rate; CRP: C-reactive protein; ANA: antinuclear antibodies; DNA ab (DS): anti-double-stranded DNA antibodies; RNP: ribonucleoprotein antibodies; Scl 70: anti-topoisomerase I antibodies; JO1: anti-JO 1 antibodies; SAA: serum amyloid A; HLA-B27: human leukocyte antigen B27.

Lab parameters (unit)		Values		References		Lab parameters (unit)				Values		References	
														Hemoglobin (g/dL)		9.9		11.5-13.5		Urine analysis				Clear			
MCV (fL)		74.6		75-87		Blood culture				Negative																	
														MCH		23.8		24-30		Autoimmune profile		ANA		Negative			
RNP		Negative																									
WBC (µL/mm³)		16.8		5.0-14.5		DNA Ab (DS)		Negative																			
						Scl 70		Negative																			
Neutrophils (%)		72.2		32-54		JO1		Negative																			
						Centromere		Negative																			
Fibrinogen (g/L)		5.85		1.3-4.1		Calprotectin (µg/g)				146.6		<15.6-50	
														CRP (mg/dL)		110		<1.0		SAA (mg/L)				1580		⩽6.4	
ESR (mm/hr)		54		1-20		HLA-B27				Negative																	

Initially, she was managed with ceftriaxone and fluids in the hospital, and three days later, she was discharged on cefixime and paracetamol. One week later, on the follow-up, she reported one episode of fever measured as 38°C, and the persistent pain of the plantar side of the left foot was present. At the time of this visit, she was clinically diagnosed as FMF and started on colchicine 0.5 mg daily. On the follow-up visit, there was improvement of her symptoms, and the fever had subsided.

## Discussion

FMF is a monogenic autoinflammatory disease that usually presents as episodic fever lasting from one to three days [3]. The fever can subside and recur every one to eight weeks [4]. A minority of patients can present with fever solely; however, the majority have generalized abdominal pain due to peritonitis (86%-92%), and joint symptoms like arthralgia (45%-64%) [3]. In our case, she had a prolonged fever that exceeded a week with limited symptom-free periods and resistance to antipyretics, representing an atypical presentation of FMF. The abdominal pain presented very late, so she did not have typical symptoms suggesting FMF. Two criteria have been introduced recently to diagnose FMF, including the Tel Hashomer criteria and the Yalcinkaya-Ozen criteria [5]. The Yalcinkaya-Ozen criteria are based on a study conducted on genetically confirmed Turkish pediatric patients with homozygous MEFV gene [5]. The purpose is to diagnose pediatric patients who are unable to undergo genetic testing or who have had inconclusive genetic findings. The criteria state that the fulfillment of two out of five components is required for the diagnosis. The presence of two or more criteria has a sensitivity of 86.5% and specificity of 93.6% [5]. In our patient, two criteria were fulfilled, including fever attacks lasting >72 hours (>38°C) and a positive family history.

In pediatrics, a fever of unknown origin (FUO) with no specific symptoms should be followed by investigations. Children with unspecified findings on physical examination and vital parameters within normal range may undergo only blood tests, as well as basic imaging such as a chest X-ray or abdominal ultrasound. In some cases, further investigations such as procalcitonin (PCT), immunoglobulins, ferritin, uricemia, muscular enzymes, Mantoux testing, and pharyngeal swabs are required [6]. A Korean case report on FMF with persistent fever started with FUO workup, including imaging, serology, and autoimmune profile, due to low clinical suspicion of FMF; all the results were negative [7]. If an autoimmune disorder is suspected, specific autoimmune markers must be assessed accordingly, such as antinuclear antibody (ANA) [6]. In our patient, the initial common investigations were negative. Further imaging modalities were also done, including abdominal ultrasound to exclude inflammatory causes. An autoimmune profile was also performed to exclude juvenile idiopathic arthritis (JIA) and other common pediatric autoimmune conditions.

The inflammatory markers were the most important initial results, as they were markedly elevated, indicating an ongoing inflammation. SAA is an essential biomarker to assess the presence and degree of FMF inflammation. Moreover, it is considered a more accurate inflammatory marker to detect a subclinical persistent inflammation compared to CRP or ESR [8].

The choice of treatment in this case was to start colchicine as soon as the clinical diagnosis of FMF was established. The recommended starting dosage of colchicine in FMF is ≤0.5 mg/day for children <5 years of age [9]. Colchicine can also be used as a diagnostic trial if the genetic analysis is unavailable or inaccessible, particularly in high-risk regions such as the Mediterranean countries [10]. In a recent case report on FMF in Korea, they started colchicine treatment initially and noticed improvement, then performed the genetic testing, which revealed mutations in the MEFV gene [7]. In our case, the patient was unable to undergo the genetic testing; therefore, based on a review done, a trial of colchicine can confirm the diagnosis of FMF if the symptoms resolve within six months and recur upon cessation [11]. We have initiated a 0.5 mg/day exact dose as a diagnostic and therapeutic trial, as genetic testing is not an option in this case. The unavailability of the genetic testing for the MEFV gene should not delay the management with colchicine if the diagnostic criteria of FMF are supportive [12].

## Conclusions

This case demonstrates an atypical presentation of FMF characterized by long-lasting fever resistant to antipyretics, as well as minimal classical serosal involvement, posing a great diagnostic challenge. Therefore, maintaining a high level of suspicion is crucial in children with recurrent febrile episodes or FUO with known suspicious ethnicity and familial history related to FMF, or unavailable genetic testing. With these atypical features presented by our patient and the absence of genetic confirmation, it significantly underscores the diagnostic complexity of FMF presentations in early childhood. Thus, early recognition and timely initiation of colchicine therapy are essential to prevent serious long-term complications, such as secondary amyloidosis.
